# Dog ownership may promote cardiometabolic health in U.S. military veterans

**DOI:** 10.1038/s41598-023-38038-4

**Published:** 2023-07-08

**Authors:** Steven H. Woodward, Stephen R. Baldassarri, Robert H. Pietrzak

**Affiliations:** 1 National Center for PTSD, Dissemination and Training Division, VA Palo Alto Healthcare System, 3801 Miranda Ave, Palo Alto, CA 94304 USA; 2 Clinical Neurosciences Division, National Center for PTSD, VA Connecticut Healthcare System, 950 Campbell Ave, West Haven, CT 06516 USA; 3 Department of Psychiatry, Yale School of Medicine, New Haven, CT 06511 USA; 4 Department of Social and Behavioral Sciences, Yale School of Public Health, New Haven, CT 06511 USA; 5 Section of Pulmonary, Critical Care and Sleep Medicine, Department of Internal Medicine, Yale School of Medicine, New Haven, CT 06511 USA; 6 Program in Addiction Medicine, Yale School of Medicine, New Haven, CT 06511 USA

**Keywords:** Cardiology, Health care, Risk factors

## Abstract

Dog ownership has been associated with reduced cardiovascular and all-cause mortality in civilian epidemiological samples. Associations between dog ownership and cardiometabolic disease were examined in the 2019–2020 wave of the National Health and Resilience in Veterans Study. Dog and cat ownership data were obtained from 3078 Veterans and cross-tabulated with self-reported, professionally diagnosed, heart disease, heart attack, stroke, high blood pressure, diabetes, and high cholesterol. In unadjusted tests, dog ownership was associated with lower rates of heart disease, high blood pressure, diabetes, and high cholesterol, while cat ownership was not. Relative to non-owners, dog owners were younger, were more likely to screen positive for posttraumatic stress disorder and/or major depressive disorder, and more active. Binary logistic regression models of associations between dog ownership and cardiometabolic disease were adjusted for age, sex, trauma load, mood disorder, substance abuse, nicotine abuse, and exercise. After adjustment, dog ownership was still associated with lower odds of hypertension and high cholesterol. Dog ownership also interacted with exercise to lower odds of heart disease and attenuated the effect of trauma load on hypertension. Conversely, age interacted with dog ownership such that odds of diabetes and stroke were higher in older Veterans who owned dogs.

## Introduction

The evidence is now strong that individuals diagnosed with posttraumatic stress disorder (PTSD) exhibit elevated rates of cardiovascular disease (CVD)^1–10^ and adult-onset Type 2 diabetes mellitus^11,12^. Researchers are now investigating candidate mechanisms. In one recent study, individuals diagnosed with PTSD were found to exhibit decreased cardiac contractility despite concurrently increased sympathetic tone as indexed by pre-ejection period^13^. In rats exposed to predator stress, Rorabaugh and colleagues observed hypersensitivity to cardiac ischemia^14–18^. Inflammation has been proposed to mediate between PTSD and insulin resistance in young military Veterans^19^. Interest is also emerging in whether and how amelioration of PTSD might reduce risk for CVD^20,21^ and diabetes^22^. Natural targets are behavioral risk factors for CVD known to be elevated in PTSD such as smoking, alcohol abuse, poor diet, and physical inactivity^11,23–31^. One proposed complementary and alternative intervention for PTSD with popular support is dog ownership^32^, which has been shown to be associated with enhanced cardiac health in the general population. Using Sweden's Register of the Total Population, Mubanga and colleagues^33^ crossed the records of all persons aged between 40 and 80 in 2001 with a registry of all dogs owned at that time. After adjusting for multiple covariates in a sample of 3,432,153, and assuming a dog lifespan of 10 years, they found support for the possibility that dog ownership was associated with reduced CVD-related mortality (acute myocardial infarction + heart failure + ischemic stroke + hemorrhagic stroke; hazard ratio = 0.77) over a 12-year follow-up period. Risk reductions were larger in persons living alone. A follow-up study by Mubanga et al^34^ found a similar pattern of risk reduction in a large sample of persons (n = 181 $,$ 696) followed for 12 years after a first myocardial infarction. Kramer et al^35^ have reviewed smaller studies yielding similar results.

The current study examined whether benefits of dog ownership on CVD and diabetes extend to US military Veterans, a population that is substantially older than their civilian counterparts^36^, predominantly male, with elevated rates of CVD, diabetes, and psychiatric and substance use disorders^37^. Cat ownership was also considered as a control.

## Methods

**Figure 1:**
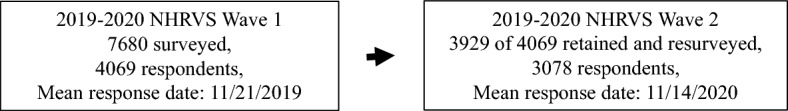
Flow chart of 2019–2020 NHRVS survey waves.


Data were obtained from the National Health and Resilience in Veterans Study (NHRVS), which comprises multiple online surveys of nationally representative samples of US military Veterans. Inaugurated in 2011, the NHRVS has recruited three independent cohorts of US Veterans from the KnowledgePanel survey panel of more than 50 $,$ 000 US households. KnowledgePanel methods employ multiple strategies to minimize bias, including (1) invited rather than self-selected respondents, (2) use of the Delivery Sequence File of the U.S. Postal Service to create a maximal frame for randomized invitation, (3) inclusion of households without internet access who are then provided with such access to enable unimodal surveying, and (4) inclusion of under-researched populations such as young adults, the rurally-domiciled, and disabled persons. Data for this study were drawn from the most recent 2019–2020 cohort. Figure 1 diagrams the sampling process. A total of 7,860 Veterans were invited to participate in the Wave 1 survey and 4,069 completed it (51.8% participation rate, mean response date: 11/21/2019). In the Wave 2 survey, which included the pet ownership questions below, 3,929 of retained Wave 1 respondents were resurveyed with 3,078 (75.6%) completing (median response date: 11/14/2020). Post-stratification weights were computed by the Ipsos statistical team to harmonize the sample distribution with that of Veterans in the 2019 Veterans Supplement of the U.S. Census Current Population Survey. Prior NHRVS studies have considered a wide range of topics including Veterans' physical health^1,2^, psychological health^38–40^, aging^38^, and suicidality^41^. Mean (SD) age of the sample was 62.6 (15.4) years (range, 23–100 years) and 9.7% were female. The pet ownership questions were embedded in a section covering mental health outcomes^41^.

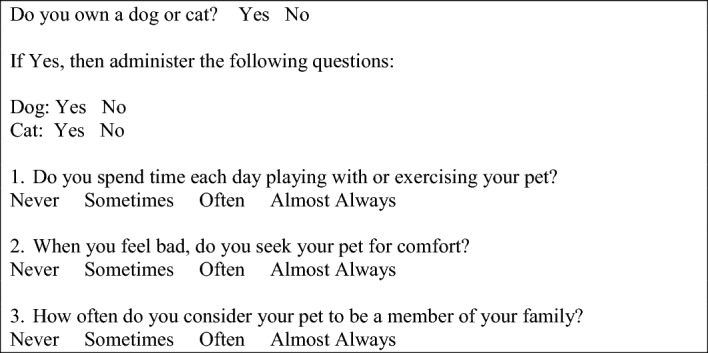


Selected cardiometabolic disease states were assessed as part of a Medical Conditions Checklist adapted from a previous population-based epidemiologic study^39^. They included heart disease, heart attack, high cholesterol, hypertension, stroke, and diabetes. The following is a sample item: “Has a doctor or healthcare professional ever told you that you have any of the following medical conditions? Heart disease?” While we refer to "diabetes" without specification of etiology or age of onset, it should be noted that a diagnosis of diabetes is an exclusion for service in the US military. The presence/absence of lifetime PTSD was assessed using the PTSD Checklist for DSM-5^42^. Lifetime major depressive disorder (MDD), alcohol use disorder (AUD), and substance use disorder (SUD) were assessed using a modified, self-report version of the MINI Neuropsychiatric Interview^43^. Psychiatric disorder variables were grouped and dichotomized as follows: PTSD and/or MDD vs. neither, and AUD and/or SUD vs. neither. Adjustment for nicotine use disorder (NUD) was based upon responses to the Fagerström Test for Nicotine Dependence^44^. Cumulative lifetime trauma was estimated by summing the number of potentially traumatic direct and indirect exposures endorsed on the Life Events Checklist for DSM-5^45^. Physical exercise habits were assessed using the Godin Leisure-Time Exercise Questionnaire (GLTEQ)^46^ which assesses typical weekly frequency of engagement in "strenuous", "moderate", versus "mild/light" exercise. The frequency of exercise within each level is estimated, multiplied by a constant (9, 5, or 3, respectively), and summed to create a total weekly exercise score which is then categorized as "Active" (24 or higher), "Moderately Active" (14 to 23), or "Sedentary/Insufficiently Active" (less than 14). Complete data for all study variables were available for 3,061 (99.4%) Veterans. Missing data were deleted case-wise within analyses.

Data analyses were conducted using SPSS v27. Chi-squares, independent-samples t-tests, and Mann–Whitney U tests were used to compare the crude rates of cardiometabolic disease states by dog and cat ownership status. A series of multivariable binary logistic regressions were then used to examine the adjusted odds of each cardiometabolic disease state across those who did and did not own dogs. The risk stratifiers, which were force-entered, were age, sex, number of direct and indirect traumas, lifetime PTSD/MDD, lifetime AUD/SUD, current NUD, and habitual exercise level (via continuous scoring of the GLTEQ). Insofar as a full model would have yielded 247 interaction terms, we employed Wald forward selection (p-value criterion for entry = 0.05) to identify statistically significant interactions. Interpretation was limited to significant two-way interactions between dog ownership and risk stratifiers. Statistically significant interactions of dog ownership and moderating variables (e.g., habitual exercise) were illustrated by computing and plotting predicted probabilities.

### Ethics statement

These secondary analyses were performed as "not human subjects research" per the Institutional Review Board of the VA Connecticut Health Care System.

## Results

Dog and cat ownership questions were answered by 99.4% and 99.3% of respondents, respectively. Dog ownership was endorsed by 1106 Veterans (39.2%) and denied by 1955 Veterans (60.8%). Cat ownership was endorsed by 723 Veterans (24.6%) and denied by 2260 Veterans (74.8%). As shown in Table 1, the unadjusted prevalence of four of the six assessed cardiometabolic disease states differed by dog ownership. Dog owners had lower prevalences of heart disease (9.5% vs. 14.4%; χ^2^ = 15.95, *p *< 0.001), high blood pressure (41.5% vs. 54.4%; χ^2^ = 48.18, *p *< 0.001), diabetes (16.0% vs. 20.8%; χ^2^ = 10.59, *p *= 0.001), and high cholesterol (38.6% vs. 49.1%, χ^2^ = 32.24, *p *< 0.001), but not lower incidences of heart attack (6.0% vs. 6.8%; χ^2^ = 0.87, *p *= 0.35) or stroke (3.6% vs. 4.2%; χ^2^ = 0.58, *p *= 0.44). In contrast to dog ownership, as shown in Table 2, cat ownership was not associated with prevalence/incidence of any of the assessed cardiometabolic disease states (heart disease: 13.0% vs. 12.9%, χ^2^ = 0.003, *p *= 0.955; heart attack: 6.9% vs. 6.2%; χ^2^ = 0.352, *p *= 0.553; stroke: 3.0% vs. 3.1%; χ^2^ = 0.018, *p *= 0.894, high blood pressure: 50.6% vs. 52%; χ^2^ = 0.289, *p *= 0.591; diabetes: 21.0% vs. 19.7%; χ^2^ = 0.616, *p *= 0.432; and high cholesterol: 45.1% vs. 46.9%; χ^2^ = 0.707, *p *< 0.400). Associations with cat ownership were not analyzed further.

**Table 1:** Prevalence of cardiometabolic disease states by dog ownership status.

	Dog owner N = 1,106	Non-dog owner N = 1,955	Pearson chi-square
	N (weighted %)	N (weighted %)	
Heart disease***	138 (9.5%)	338 (14.4%)	15.95, *p *< 0.001
Heart attack	83 (6.0%)	155 (6.8%)	0.87, *p *= 0.35
Stroke	46 (3.6%)	94 (4.2%)	0.58, *p *= 0.44
High blood pressure***	531 (41.5%)	1,114 (54.4%)	48.18, *p *< 0.001
Diabetes**	216 (16.0%)	458 (20.8%)	10.59, *p *= 0.001
High cholesterol***	488 (38.6%)	1,043 (49.1%)	32.24, *p *< 0.001

Significant association: **p < 0.01; ***p < 0.001.

**Table 2:** Prevalence of cardiometabolic disease states by cat ownership status.

	Cat owner N = 723	Non-cat owner N = 2,260	Pearson chi-square
	N (weighted %)	N (weighted %)	
Heart disease	94 (13.0%)	292 (12.9%)	0.003, *p *= 0.995
Heart attack	45 (6.9%)	155 (6.2%)	0.352, *p *= 0.553
Stroke	22 (3.0%)	71 (3.1%)	0.018, *p *= 0.894
High blood pressure	366 (50.6%)	1,170 (52.0%)	0.289, *p *= 0.591
Diabetes	152 (21.0%)	455 (19.7%)	0.616, *p *= 0.432
High cholesterol	326 (45.1%)	1,060 (46.9%)	0.707, *p *= 0.400

Significant association: **p < 0.01; ***p < 0.001.

As shown in Table 3, relative to non-dog owners, dog owners were younger (mean age 57.7 vs. 65.8 years, Mann–Whitney U = 6,814,40, *p *< 0.001), more likely to be female (14.1%, vs. 7.9%, χ^2^ (1) = 20.9, *p *< 0.001), and more likely to report traumatic events (mean of 10.3 vs. 8.2, Mann–Whitney U = 1,133,053, *p *< 0.001). They were also more likely to screen positive for lifetime PTSD/MDD (30.0% vs. 18.9%, χ^2^ (1) = 45.8, *p *< 0.001), AUD/SUD (45.8% vs. 41.7%, χ^2^ (1) = 5.0, *p *= 0.03), and NUD (18.4% vs. 15.4%, χ^2^ (1) = 4.5, *p *= 0.03). Dog owners also reported more physical exercise than non-dog-owners (GLTEQ mean = 36.0 vs. 29.9, Mann–Whitney U = 1,035,538, *p *< 0.001).

**Table 3:** Prevalence of cardiovascular disease-relevant measures by dog ownership status.

	Dog owner	Non-dog owner	Test of difference	*p*
Mean Age	57.7	65.8	M-W U = 681,440	< 0.001
Female	14.1%	7.9%	χ^2^ = 23.9	< 0.001
Mean # Traumas	10.3	8.2	M-W U = 1,133,053	< 0.001
Lifetime PTSD/MDD	30.0%	18.9%	χ^2^ = 45.8	< 0.001
Lifetime AUD/SUD	45.8%	41.7%	χ^2^ = 5.0	= 0.03
Lifetime NUD	18.4%	15.4%	χ^2^ = 4.5	= 0.03
Mean GLTEQ	36.0	29.9	M-W U = 1,035,538	< 0.001
Mean BMI	29.4	29.1	t = 1.7	= 0.087

Table 4 summarizes binary logistic regression models adjusted for the selected risk stratifiers. These revealed that dog ownership remained associated with significantly lower odds of high cholesterol (21% lower odds, 95%CI = − 0.33 to − 0.07, *p *< 0.01) and high blood pressure (16% lower odds, 95%CI = − 0.29–− 0.01, *p *< 0.05), but not with the other outcomes.

**Table 4:** Results of logistic regression models examining association between dog ownership and cardiovascular disease-relevant measures.

	Heart disease	Heart attack	Stroke	High blood pressure	Diabetes	High cholesterol
Nagelkerke R^2^	0.162	0.132	0.066	0.150	0.116	0.092
	OR (95%CI)	OR (95%CI)	OR (95%CI)	OR (95%CI)	OR (95%CI)	OR (95%CI)
Dog ownership	0.96 (0.74–1.24)	1.28 (0.92–1.79)	1.37 (0.90–2.07)	0.84 (0.71–0.99)*	0.93 (0.75–1.16)	0.79 (0.67–0.93)**
Age	1.06 (1.05–1.07)***	1.06 (1.04–1.08)***	1.04 (1.02–1.06)***	1.04 (1.04–1.05)***	1.04 (1.03–1.05)***	1.03 (1.02–1.04)***
Male gender	2.41 (1.13–5.15)*	5.80 (1.30–25.83)*	1.97 (0.64–6.02)	1.48 (1.09–2.00)*	0.94 (0.62–1.43)	1.26 (0.93–1.70)
Traumas	1.02 (1.01–1.04)**	1.03 (1.02–1.05)***	0.99 (0.97–1.02)	1.02 (1.01–1.03)**	1.02 (1.01–1.04)***	1.02 (1.01–1.03)**
Lifetime MDD/PTSD	1.04 (0.74–1.45)	1.32 (0.87–2.000	1.12 (0.66–1.91)	0.94 (0.76–1.16)	0.98 (0.75–1.29)	1.03 (0.84–1.27)
Lifetime AUD/SUD	0.86 (0.67–1.11)	0.85 (0.61–1.18)	1.29 (0.85–1.95)	1.10 (0.93–1.31)	1.03 (0.84–1.27)	0.87 (0.73–1.03)
Lifetime NUD	1.90 (1.43–2.53)***	1.88 (1.31–2.70)**	1.55 (0.98–2.46)	1.42 (1.14–1.77)**	1.46 (1.14–1.88)**	1.33 (1.07–1.64)*
Exercise level	0.83 (0.70–0.99)*	0.84 (0.67–1.05)	0.68 (0.49–0.96)*	0.98 (0.90–1.06)	0.60 (0.51–0.71)***	0.87 (0.79–0.95)**
Dog ownership x Exercise level	0.67 (0.45–0.98)*	–	–	–	–	–
Dog ownership x Traumas	–	–	–	0.97 (0.96–0.99)*	–	–
Dog ownership x Age	–	–	1.05 (1.01–1.08)*	–	1.03 (1.01–1.04)**	–
Dog ownership x Lifetime NUD	–	–	2.57 (1.02–6.23)*	–	–	–

Statistically significant: *p < 0.05; **p < 0.01; ***p < 0.001.

OR = odds ratio; 95%CI = 95% confidence interval; MDD = major depressive disorder; PTSD = posttraumatic stress disorder; AUD = alcohol use disorder; SUD = substance use disorder; NUD = nicotine use disorder.

Model fit (X^2^ and p): Heart disease = 263.66, < 0.001; Heart attack = 152.85, < 0.001; Stroke = 54.72, < 0.001; High blood pressure = 31.96, < 0.001; Diabetes = 220.79, < 0.001; High cholesterol = 209.66, < 0.001.

Models included all of the specified variables, as well as all possible 2-way interaction terms; only statistically significant interaction terms are shown.

Nagelkerke R^2^ values are computed from models that included all independent variables and significant interaction terms.

**Figure 2:**
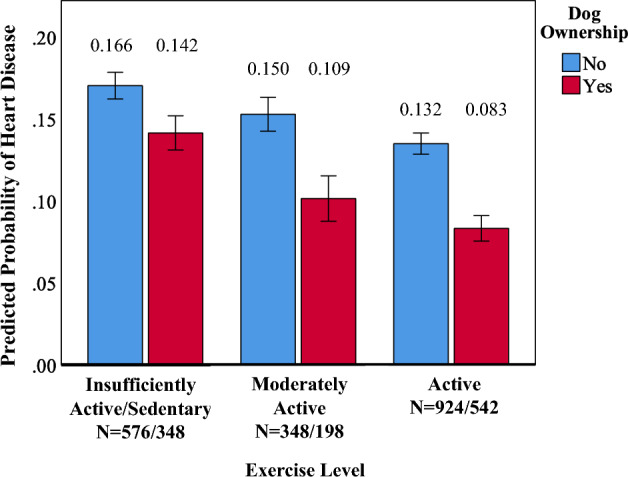
Plot of the interaction of dog ownership and exercise level on the probability of endorsement of heart disease. Probabilities are adjusted for dog ownership, age, sex, number of direct and indirect traumas, lifetime PTSD/MDD, lifetime AUD/SUD, current NUD, and habitual exercise level.

**Figure 3:**
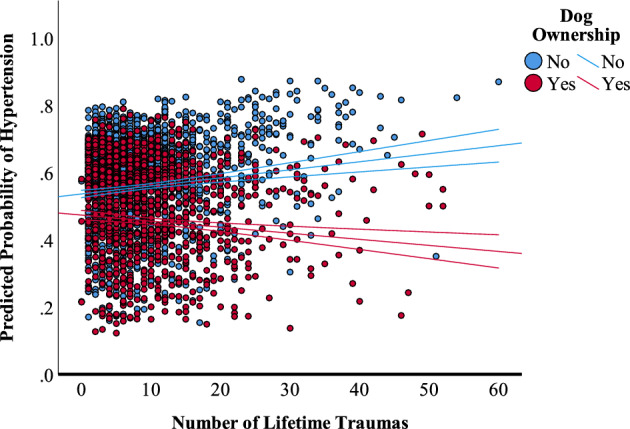
Plot of the interaction of dog ownership and trauma load on the probability of endorsement of hypertension. Probabilities are adjusted for dog ownership, age, sex, number of direct and indirect traumas, lifetime PTSD/MDD, lifetime AUD/SUD, current NUD, and habitual exercise level.

Significant two-way interactions were also observed. Specifically, dog ownership and exercise level interacted constructively so that as habitual exercise increased, the odds of heart disease in dog owners decreased relative to non-dog owners (odds ratio [OR] = 0.67, 95%CI = 0.45–0.98, *p *= 0.042; See Fig. 2). Further, as the number of lifetime traumatic events increased, dog owners had significantly lower odds of high blood pressure relative to non-dog owners (OR = 0.97, 95%CI = 0.96–0.99, *p *= 0.013; See Fig. 3).

**Figure 4:**
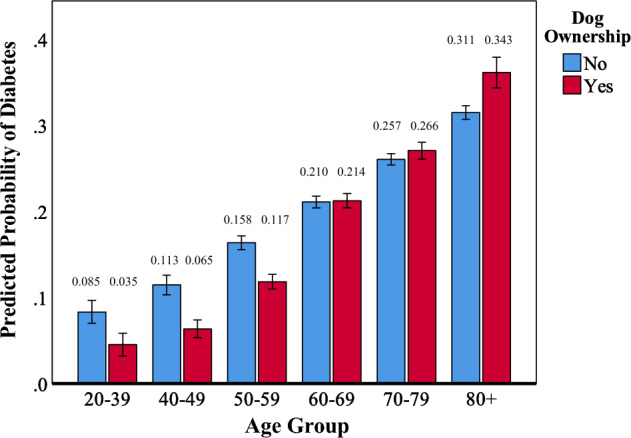
Plot of the interaction of dog ownership and age on the probability of endorsement of diabetes. Error bars represent 95% confidence intervals. Probabilities are adjusted for dog ownership, age, sex, number of direct and indirect traumas, lifetime PTSD/MDD, lifetime AUD/SUD, current NUD, and habitual exercise level. Cell sizes: Non-Dog Owners: 20–39 = 43; 40–49 = 87; 50–59 = 233; 60–69 = 440; 70–79 = 834; 80 +  = 318. Dog Owners: 20–39 = 64; 40–49 = 106; 50–59 = 205; 60–69 = 328; 70–79 = 334; 80 +  = 69.

**Figure 5:**
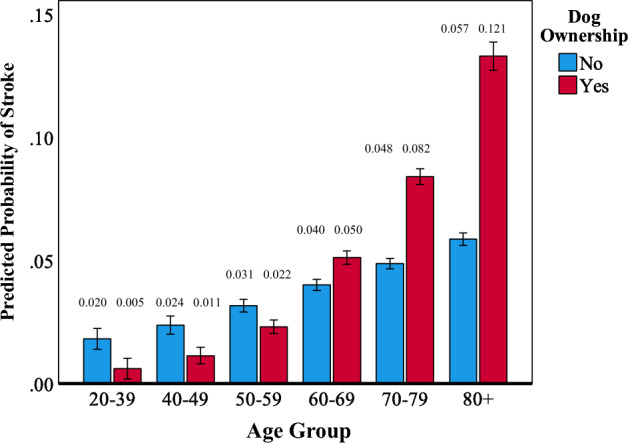
Plot of the interaction of dog ownership and age on the probability of endorsement of stroke. Error bars represent 95% confidence intervals. Probabilities are adjusted for dog ownership, age, sex, number of direct and indirect traumas, lifetime PTSD/MDD, lifetime AUD/SUD, current NUD, and habitual exercise level. Cell sizes: Non-Dog Owners: 20–39 = 43; 40–49 = 87; 50–59 = 233; 60–69 = 440; 70–79 = 834; 80 +  = 318. Dog Owners: 20–39 = 64; 40–49 = 106; 50–59 = 205; 60–69 = 328; 70–79 = 334; 80 +  = 69.

**Figure 6:**
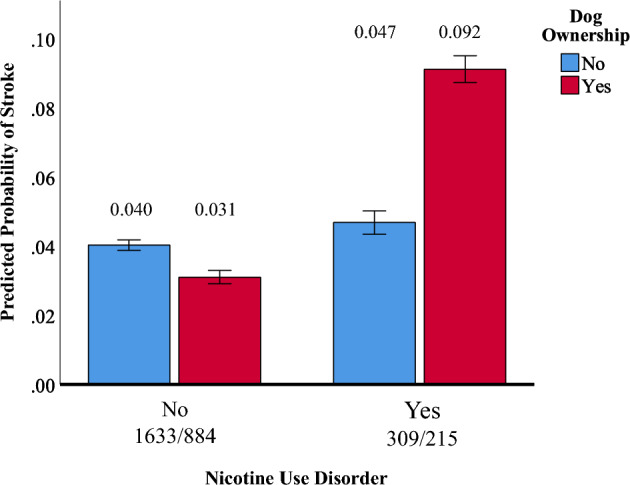
Plot of the interaction of dog ownership and nicotine use disorder on the probability of endorsement of stroke. Error bars represent 95% confidence intervals. Probabilities are adjusted for dog ownership, age, sex, number of direct and indirect traumas, lifetime PTSD/MDD, lifetime AUD/SUD, current NUD, and habitual exercise level.

Interactions suggestive of negative associations between dog ownership and indices of cardiometabolic health were also observed. A significant age by dog ownership interaction was observed for diabetes (OR = 1.03, 95%CI = 1.01–1.04, *p *< 0.005). As shown in Fig. 4, the apparently protective effect of dog ownership disappeared after age 70 and even reversed in Veterans over 80. Similarly for stroke, the apparent protective effect of dog ownership disappeared after age 60 and reversed in Veterans over 70 (OR = 1.05, 95%CI, 1.01–1.08, *p *= 0.015; See Fig. 5). Finally, while dog ownership appeared protective against stroke in those without NUD, the opposite was observed in those with NUD (OR = 2.57, 95%CI = 1.02–6.23, *p *= 0.044; See Fig. 6).

## Discussion

The results of this study of a nationally representative sample of U.S. military Veterans suggest that dog ownership may be associated with lower odds of high blood pressure and high cholesterol in this population. In contrast, cat ownership was not associated with differential prevalence/incidence of any of the disease states tested, replicating Enmarker et al^47^. Dog ownership also interacted with elevated habitual exercise to yield reduced odds of heart disease. The source of this constructive interaction is difficult to resolve further without more precise estimates of exercise. For example, Zhou et al^48^ recently observed a curvilinear relationship between an estimate of total daily activity, including commuting and work-related activity, and CVD incidence in a large sample of hypertensive adults. Mixed exercise has been promoted by the American Heart Association as reducing CVD risk^49^; but, without a comprehensive tally of exercise types from participants, we can only speculate that the addition of dog walking may have yielded a mixed exercise program in this sample. It is interesting in this regard that Mubanga et al^33^ found that ownership of "hounds" conferred the greatest CVD risk reduction in their sample, while Pickup et al^50^ found that "gundogs" were the breed group most frequently walked in their sample of more than 12 $,$ 000 pet dogs. The extension of the interaction observed here into the most active category suggests that the benefits associated with dog ownership were not limited to increased walking. A testable hypothesis is that the increased walking associated with dog ownership may function as a gateway to engagement in more vigorous exercise, which may in turn confer additional protection against cardiometabolic disease.

Dog-ownership also moderated the association between trauma load and high blood pressure, rendering it slightly negative. Associations between both trauma and PTSD and hypertension have been observed in both cross-sectional and longitudinal studies^28,29,39,51–53^; however, the largest study to date, a trans-ethnic meta-analysis of more than 70 $,$ 000 participants that included genetic markers of risk found only small associations between PTSD symptomology and blood pressure. Moreover, those associations differed directionally over different cohorts and for systolic versus diastolic blood pressures^54^. Further research is necessary to disentangle mechanisms underlying the association between cumulative trauma load, dog ownership, and hypertension risk.

In addition to the apparent benefits noted above, conditional risk increases for certain cardiometabolic conditions were also observed in association with dog ownership. As expected, the probability of self-reported diagnoses of both diabetes and stroke increased with age; but though dog ownership was associated with lower likelihood of both outcomes through mid-life, these effects reversed in the eighth and ninth decades, respectively. Laine et al^55^ also observed higher odds of diabetes in a sample of older males (but not females) who owned dogs relative to those who did not. Dog ownership also interacted with NUD to increase the risk of stroke independent of age. It is possible that both advanced age and nicotine use may operate to nullify the relationship between dog ownership and physical activity. Mobility declines in advanced age due to the accumulation of musculoskeletal injuries and diseases^56^. As well, smokers are less active than non-smokers^57,58^. That said, if the activity-related benefits of dog ownership are gated by advanced age and smoking, they should disappear in those conditions rather than reversing to augment risk. Detailed investigation of the lifestyles and family circumstances of the respondents in their eighth and ninth decades could reveal factors that may interact with dog ownership to increase risk for diabetes and stroke. As regards the doubling of self-reported stroke incidence in dog owners who smoked, we may need to consider whether increased activity could interact with smoking to adversely impact cardiac health in the elderly. While such an interaction appears unlikely, the large literature addressing lifestyle factors and stroke is generally silent on interactions between exercise and smoking, even though their respective main effects are well-established^59,60^. Such an interaction could involve a third mediating condition, such as atrial fibrillation, which can be triggered by both exercise and smoking, and is a potent risk factor for stroke^61^. Assessment of conditions possibly potentiated by adverse interactions of exercise and smoking should be included in future surveys addressing the cardiovascular benefits of dog ownership. As well, because survey methods are limited to those still alive, nonsurvey methods, such as registries, would be advantageous in allowing for the measurement of both fatal and nonfatal events. Notwithstanding the apparent benefits observed, we must remain alert to the possibility that a non-obvious consequence or covariate of dog ownership could interact with smoking to increase the incidence of stroke.

Limitations of this study include its cross-sectional design which cannot rule out the possibility that an advantageous cardiac risk profile characterized those who elected to obtain dogs, rather than resulting from dog ownership, itself. Further, analyses of cardiac risk profiles among those who acquired dogs since the last wave of the NHRVS, a period during which many people acquired dogs to mitigate the adverse social impacts of the COVID-19 pandemic, may shed more light on the possibility that dog ownership could play a causative role in cardiometabolic disease risk reduction. A surprising feature of the results was that the PTSD/MDD factor was not associated with elevated cardiometabolic disease. This may be due to the older mean age of the sample (62.6 years)^62^. Finally, the explanatory power of the multivariable regression models was relatively low, ranging from 0.066 to 0.162. More research using a broader range of potential explanatory variables and nuanced measures (e.g., frequency and types of exercise with dogs) is needed to further understand the role of dog ownership in relation to cardiometabolic health.

## Conclusion

Dog ownership may be associated with lower odds of hypertension and high cholesterol in U.S. military Veterans, and, when combined with exercise, associate with reduced heart disease; however, some of the protective effects appear to be moderated or even reversed by advanced age and nicotine use disorder. While dog ownership could provide an adjunctive, non-pharmacologic avenue for primary and secondary prevention of PTSD-associated cardiometabolic disease, further research may identify conditions in which it is contraindicated.

## Data Availability

The data that support the findings of this study are available on request from R. H. P. The data are not publicly available due to privacy or ethical restrictions.
